# Similarity of the dog and human gut microbiomes in gene content and response to diet

**DOI:** 10.1186/s40168-018-0450-3

**Published:** 2018-04-19

**Authors:** Luis Pedro Coelho, Jens Roat Kultima, Paul Igor Costea, Coralie Fournier, Yuanlong Pan, Gail Czarnecki-Maulden, Matthew Robert Hayward, Sofia K. Forslund, Thomas Sebastian Benedikt Schmidt, Patrick Descombes, Janet R. Jackson, Qinghong Li, Peer Bork

**Affiliations:** 1 Structural and Computational Biology Unit, European Molecular Biology Laboratory, Heidelberg, Germany; 2 Nestlé Purina Research, St. Louis, MO USA; 3 Nestlé Institute of Health Sciences, Lausanne, Switzerland; 4 Max Delbrück Centre for Molecular Medicine, 13125 Berlin, Germany; 5 Department of Bioinformatics, Biocenter, University of Würzburg, 97074 Würzburg, Germany

**Keywords:** Microbiome, Diet, Metagenomics, Dog microbiome, Human microbiome, Mouse microbiome, Pig microbiome

## Abstract

**Background:**

Gut microbes influence their hosts in many ways, in particular by modulating the impact of diet. These effects have been studied most extensively in humans and mice. In this work, we used whole genome metagenomics to investigate the relationship between the gut metagenomes of dogs, humans, mice, and pigs.

**Results:**

We present a dog gut microbiome gene catalog containing 1,247,405 genes (based on 129 metagenomes and a total of 1.9 terabasepairs of sequencing data). Based on this catalog and taxonomic abundance profiling, we show that the dog microbiome is closer to the human microbiome than the microbiome of either pigs or mice. To investigate this similarity in terms of response to dietary changes, we report on a randomized intervention with two diets (high-protein/low-carbohydrate vs. lower protein/higher carbohydrate). We show that diet has a large and reproducible effect on the dog microbiome, independent of breed or sex. Moreover, the responses were in agreement with those observed in previous human studies.

**Conclusions:**

We conclude that findings in dogs may be predictive of human microbiome results. In particular, a novel finding is that overweight or obese dogs experience larger compositional shifts than lean dogs in response to a high-protein diet.

**Electronic supplementary material:**

The online version of this article (10.1186/s40168-018-0450-3) contains supplementary material, which is available to authorized users.

## Background

The gut microbiome has been shown to impact the health of its host, in particular by mediating the impact of diet on host body weight [1–3]. Specific interactions between dietary components, the microbiome, and the host are however still cumbersome to determine and confirm. While it is possible to perform such studies in humans, the costs involved, time constraints, and the need to control many confounders make it desirable to conduct such research in other species, where the findings might be predictive of human results. The traditional lab mouse has been widely used for this purpose, but its value has been questioned [4, 5]. Recently, pigs, although much more expensive, have been proposed as an alternative model as they may be closer to humans in phenotype and diet [6, 7]. Pigs have long been known to possess a gastrointestinal tract similar to that of humans and have been used as model animals in nutrition studies [8].

**Fig. 1:**
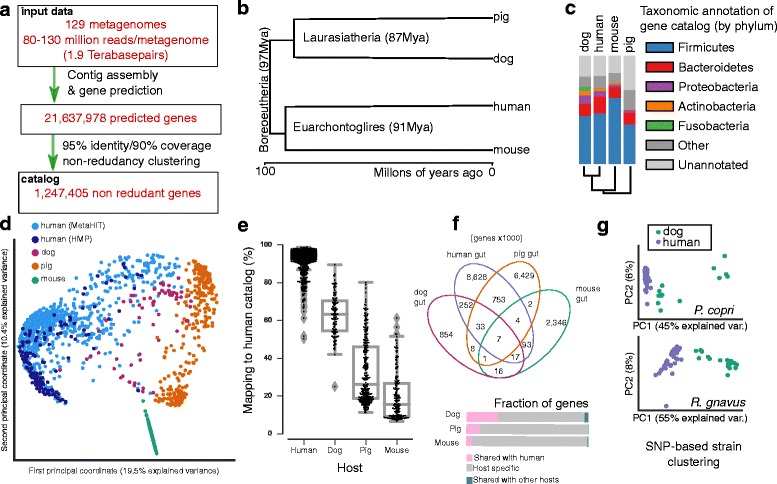
Dog gut microbiome gene catalog in comparison to human, mouse and pig. **a** Overview of gene catalog generation pipeline. **b** Phylogenetic relationship of the four hosts considered in this study, obtained by whole genome alignments, as reported by Murphy et al. [10]. **c** Distribution by phylum of the genes in the dog, human, mouse, and pig gut gene catalogs. **d** Principal coordinate analysis of genus-level taxonomic distribution in four mammal hosts (including two human cohorts), based on abundance-weighted Jaccard distance. **e** Mapping rates of reads from each of the four hosts when recruited against the human gene catalog. **f** Overlap of gene catalogs at 95% identity between the catalogs of the four species considered (in thousands of genes). **g** Principal coordinate analysis of SNP-based differentiation of strains from human and dog for the two most abundant species in dogs

Here, we leverage a nutritional study on dogs (*Canis lupus familiaris*) to study the relationship between its microbiome and those of humans, pigs, and mice. The similarity of the dog microbiome to that of humans has also been explored using taxonomic profiling (16S-amplicon-based), focusing on the effects of IBD (inflammatory bowel disease), with both similarities and differences being reported in how IBD affects the microbiome [9]. Phylogenetically, humans, dogs, pigs, and mice are at a similar genetic distance from each other (see Fig. 1b), with the last common ancestor having lived ca. 97 million years ago (Additional files 1, 2, 3, 4, and 5) [10, 11]. However, it has previously been reported that, across the mammalian kingdom, microbial composition clusters by the diet of the host [12, 13]. Dogs were domesticated early in modern human history and frequently shared food resources with humans, which has been suggested as a selective force on the dog digestive and metabolic system post-domestication [14].

Dogs are pets and companion animals and, as such, their own well-being is of intrinsic concern. This implies that extra care is necessary when studying these animals. It also implies that it is important to unravel the relationship between the dog microbiome and the health of its host [15]. In particular, an increasing prevalence of obesity in companion dogs is a large problem as it has an impact on pet health [16]. Recent estimates of overweight or obese dogs in the Western world imply that more than half of all dogs are above their ideal weight [17, 18]. As in humans, calorie restriction can be effective in reducing a dog’s weight [19, 20]. However, it often leads to emotional stress in pet owners, which in turn leads to low compliance, impairing its practical effectiveness [21]. In contrast, high-protein diets have been reported as effective as they lead to weight loss while minimizing muscle loss [22] and induce satiety when combined with high fiber [23]. In humans, low-carbohydrate diets have been similarly effective at reducing weight, at least over the short term [24, 25], with evidence that they increase satiety compared to low-calorie diets [26].

**Fig. 2:**
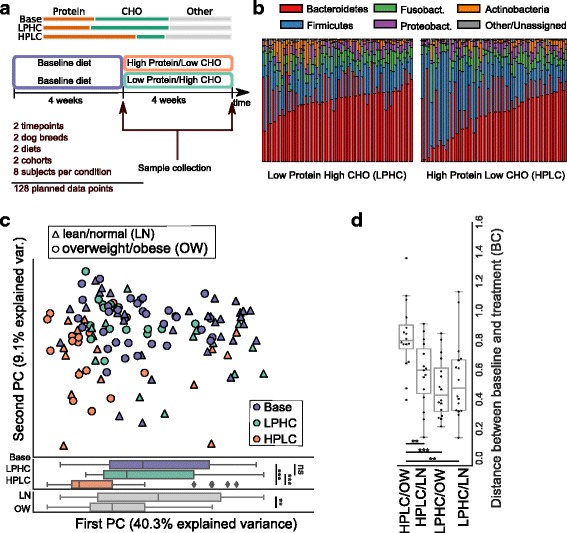
Effects of diet on the dog gut microbiome. **a** Study design (CHO carbohydrates, LPHC lower protein higher carbohydrates, HPLC high-protein low-carbohydrates). **b** Phylum-level relative abundances in the three diets; data is paired so that adjacent bars represent data from the same dog (before and after dietary intervention). **c** Principal coordinate analysis (using Bray-Curtis on log-transformed data as the underlying distance measure) based on taxonomic composition at the genus level (top panel) and the distributions of samples along the first principal component by diet and phenotype (bottom panel), *p < 0.05, **p < 0.01, ***p < 0.001; testing using Mann-Whitney-Wilcoxon test, after multiple hypothesis using the two-step Benjamini-Hochberg method; n.s. non-significant. **d** Shifts in microbiome composition vary for different diets and phenotype. The differences in relative abundance between the baseline and the post-treatment sample from the same dog, measured as Bray-Curtis (BC) distance after log-transformation (*p < 0.05, **p < 0.01, ***p < 0.001)

In this context, we investigated the effect of dietary intervention on the dog gut microbiome in a randomized control trial (RCT), containing equal numbers of lean/normal (LN) and overweight/obese dogs (OW). After a feed-in period with a baseline diet (Base), dogs were randomly assigned to one of two diets: (1) a high-protein/low-carbohydrate (HPLC) diet or a (2) lower-protein/higher-carbohydrate (LPHC) diet (Fig. 2a and Additional file 6: Table S6). This experimental design allowed us to explore differential effects of diet on the microbiome of OW dogs compared to that of LN dogs. Using shotgun deep sequencing, we built a non-redundant gene catalog of the dog gut microbiome, which we compared to previously published catalogs for the human, mouse, and pig guts.

## Results and discussion

### A dog gut microbiome gene catalog

A total of 129 dog stool samples were collected from 64 dogs (32 Labrador retrievers and 32 beagles; see Additional file 1: Table S1 for physical characteristics of the study cohort), with two samples from each dog (except for a single case where three samples were collected from the same dog). DNA was extracted and Illumina-sequenced in pair-end mode (125 bases per read). Each metagenome contains an average of 117 million paired-end reads (s.d. 32 million), leading to a total of 1.9 terabasepairs over all samples (Fig. 1a and Additional file 2: Table S2). Following previously developed approaches [27], we assembled the metagenomic reads from each sample into contigs, predicted genes on these contigs, and, finally, clustered the predicted genes from all samples into a non-redundant gene catalog (see Fig. 1a; “Methods” section; [28]). This catalog contains 1,247,405 non-redundant (at 95% nucleotide sequence identity) coding sequences, of which 630,230 (50.5%) are complete genes with an average size of 884 base pairs, compared to an average of 571 base pairs for incomplete ones.

As many as 97% of the reads can be recruited back to the catalog, indicating that the catalog already captures almost all of the genomic content in these samples (Fig. 1e). Two published dog metagenomes [29] from pooled dog fecal samples of six hound-cross dogs, sequenced using 454 technology with only ca. 500,000 reads each, were used to assess the generality of this catalog beyond the study cohort. When mapping these against our catalog using the same identity cutoff as for the catalog generation (95%), we were able to recruit 90.4 and 92.4% of reads to our catalog, for the two metagenomes, respectively. This implies that our catalog already contains most of the genomic content of the gut microbiome of dogs in a Western pet care center.

Taxonomic annotation (see the “Methods” section) showed that the dog gut microbiome gene catalog is predominantly composed of five phyla: Firmicutes, Bacteroidetes, Proteobacteria, Actinobacteria, and Fusobacteria, with the first two contributing more than half the detected genes (Fig. 1c).

### A comparison of the dog gut gene catalog with those from other mammals

We compared our gene catalog with three previously published gut microbial gene catalogs: from human [28], pig [7], and mouse [30] hosts, which had been built based on similar (Illumina sequencing) data with similar computational procedures. We applied the same taxonomic annotation to all catalogs (see the “Methods” section). The phylum-level distribution of genes in the dog gut is most similar to that of the human gut catalog, although we observe a higher proportion of genes from Fusobacteria (Fig. 1c). The mouse catalog contains the largest fraction of Firmicutes genes among the four species considered, while the pig catalog has a higher fraction of genes which cannot be annotated (Additional file 7).

As this analysis does not account for differences in abundance of genes or microbes, we compared the microbiomes using genus-level relative abundances (using abundance-weighted Jaccard as the basis for an ordination, Fig. 1d). The dog gut microbiome is closer to that of humans than the other non-human microbiomes (all pairwise comparisons are statistically significant, *p* values below computational precision limits, two-tailed Mann-Whitney-Wilcoxon test; see Additional file 8: Figure S2).

To further quantify the overlap of the three animal gut microbiomes with that of the human, we recruited short sequencing reads from each host-associated gut microbiome to the human gut gene catalog [28], accounting for gene differential abundance (Fig. 1e). As expected, human reads from the MetaHIT [31] and the HMP projects [32] mapped at the highest rate to the human catalog. Among the animal microbiomes, a much larger fraction of dog reads map to the human catalog than is the case for pigs: 63% of dog reads could be mapped to the human catalog, compared to only 32.9% of pig and 19.9% of mouse reads. When mapping human reads to the animal catalogs, 28% of reads can be mapped to the dog catalog, just slightly more than the fraction that can be mapped to the pig catalog, 27.2%. A lower rate, 22.5%, maps to the mouse catalog (Additional file 9: Figure S3).

To evaluate the overlap between the gene catalogs, we clustered all the catalogs together using the same parameters as were used when building the catalogs (see Fig. 1d). The dog gut gene pool overlaps most with the human microbiome (309,232 out of 1,247,405, circa 26%) and the murine one least (122,131 out of 2,487,431; 4.9%), with the pig catalog in-between (797,746 out of 7,238,249; 11.0%), the latter very similar to a previous report [7]. These conclusions are robust to removing low abundance genes or equalizing the number of genes by random sampling (see Additional file 10: Figure S4). Due to its larger size (9,780,814 genes), the human catalog overlaps with the animal microbiomes at much lower rates, namely 3.2% for dogs, 8.2% for pigs, and only 1.2% in the case of mouse.

The four-way intersection contains only a small number of genes (7513 out of a total of 21,385,247 genes considered). This suggests that although there are similar bacteria at the genus and even species level (Additional file 3: Table S3), most strains harbor host-specific genes [33]. To test this hypothesis and to ensure that the similarity of the dog and human microbiomes were not due to direct transmission of microbes from human to dogs, we confirmed the host specificity of strains by profiling single-nucleotide polymorphisms (SNPs) for species present in our dog samples and in publicly available human microbiome samples. Among the species with high enough coverage using default metaSNV parameters [34], only for a single species, *Bacteroides sp. D20* was a minimal overlap in SNP space observed between any human and dog strains, due to a single dog sample (the two most abundant species shown in Fig. 1f; for all six species that could be reliably profiled given the depth of sequencing, see Additional file 11: Figure S5). Thus, we conclude that persistent sharing of microbial strains between hosts of a different species is a rare event.

These different analyses consistently show that, of the three animal gut microbiomes considered, the mouse gut microbiome (the current go-to model system) is the least similar to the human gut microbiome of the three non-human animals studied. When comparing pig and dog gut microbiomes to the human one, considering in particular the analyses that are robust to the presence in the catalog of rare and low abundance genes (by taking the abundances into account), we conclude that, overall, the dog gut microbiome has a higher taxonomic and functional overlap with the human gut microbiome. As microbial gut strains are host-specific, this similarity cannot be explained solely by direct transmission between dogs and humans. Rather, it must be a function of similar physiology and lifestyle. To further explore the behavior of the dog gut microbiome, particularly in comparison to that of humans, we investigated the dog microbiome response to dietary intervention.

#### Effect of diet on the dog gut microbiome

Sixty-four dogs from two breeds were fed for 4 weeks on a common baseline diet (Base, diet details see [35]), followed by random assignment to one of two possible diet interventions: high-protein/low-carbohydrate (HPLC) or lower protein/higher carbohydrate (LPHC). The Base diet was more similar to the LPHC (Fig. 2a (top); Additional file 5: Table S5). To avoid the confounding effect of changes in the host phenotype, dogs were fed to maintain initial body weight (minimum energy requirement). Stool samples were collected before and at the end of the diet intervention (Fig. 2a). To control for possible batch effects, the study subjects were randomly split into two groups of 32 dogs and the procedure was repeated for each groups, at the same pet care center, 1 month apart. One of the dogs had to be excluded from analysis due to an antibiotic treatment for an infection unrelated to the study.

In response to the diets, we see a large shift in the overall taxonomic composition of the microbiome (Fig. 2 b–d; *p* ≤ 0.0001 using PERMANOVA [36] for diet effect; see also Additional file 12: Figure S6 which presents the distance boxplots for all samples, and Additional file 13: Figure S7, which presents the same results using Unifrac [37] and PINA [38] distances as an alternative). Specifically, the microbiome of HPLC-fed dogs shows a larger shift than that of LPHC-fed dogs, when compared to the Base diet, which is in line with the similarity between the LPHC and Base diets (Fig. 2a (top)). The consistency of the community shift argues for a direct effect of the diet as, in the absence of intervention, the dog microbiota has been reported to be stable over time, using 16S rRNA profiling [39].

In human studies, there have been several conflicting reports of the relationship of the Firmicutes:Bacteroidetes phylum ratio with obesity, with some authors reporting a higher ratio in obese individuals [40], no difference [41], or even a lower ratio [42]. For the dogs, we see a non-significant difference between overweight/obese (OW) and lean/normal (LN) dogs at the end of the baseline period, with higher Bacteroidetes in OW dogs (*p* = 0.064, two-tailed Wilcoxon test). However, we observe a large and significant difference induced by the diet, with the HPLC resulting in a higher Firmicutes:Bacteroidetes ratio in both OW and LN dogs than LPHC (Additional file 14: Figure S8).

At the genus level, the ratio of *Bacteroides* to *Prevotella* has also been found to be important in the human gut microbiome. It has been shown to change in response to diet, with higher *Prevotella* relative abundance being observed in high carbohydrate diets, while higher relative abundance of *Bacteroides* has been associated with a high protein diet [43, 44]. In our dog data, we observe that the ratio of *Prevotella* to *Bacteroides* is higher in the baseline and LPHC when compared to the HPLC (*p* = 4·10^−10^, Kruskal-Wallis test over the three diets, all pairwise comparisons are also significant, with *p* < 0.001; see Additional file 15: Figure S9), reproducing the observations in human diet studies. A differential impact of two diets differing in protein/carbohydrates on the gut microbiome of kittens was also previously reported [45, 46]. However, in that case, no global large shift was observed in the overall Firmicutes:Bacteroidetes ratio between diets, while, at the genus level, *Megasphaera* represented a large fraction of the microbiome of kittens fed an MPMC (moderate-protein/moderate-carbohydrate) diet. In the dog microbiome, this taxon represents only a small fraction of the microbiota (average relative abundance of 1.1·10^−3^), as it does in humans (average relative abundance of 2.8·10^4^).

The highest overall shift in community composition relative to pre-treatment baseline was observed in HPLC-fed OW dogs (*p* = 0.00014, two-tailed Wilcoxon test on compositional dissimilarities between baseline and post-intervention samples, comparing HPLC/OW to the rest of data; see also Fig. 2d). This effect cannot be explained by any single genus, as it remains statistically significant in every case after removing any single, pair, or triplet of genera (all tests have *p* < 0.05; two-tailed Wilcoxon test, comparing HPLC/OW to the rest of data, as above). Rather, the shift seems to be driven by a combination of four genera: *Lactobacillus*, *Prevotella*, *Streptococcus*, and *Turicibacter*, all of which showed significantly higher abundance variation in HPLC/OW dogs than in all other subcohorts. Thus, the OW dogs’ microbiome was more sensitive to the dietary shift from base to HPLC (which was a more drastic intervention than the switch to LPHC). This is consistent with the view that their microbiome resides in a less stable state compared to those of the healthy LN population [47, 48].

**Fig. 3:**
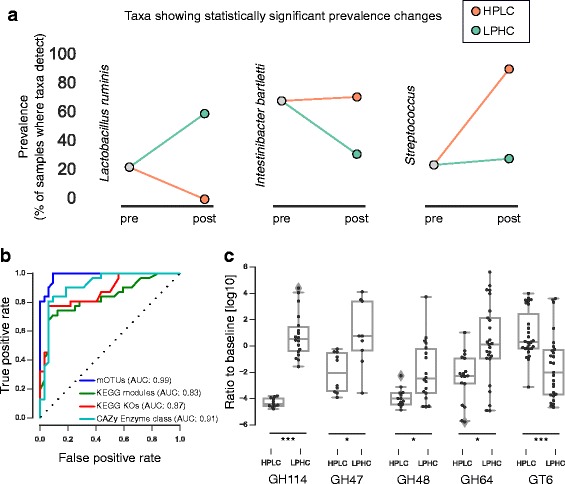
**a** Prevalence changes for three taxa showing statistically significant effects (*p* < 0.05 after multiple hypothesis testing). **b** Predictability of diet based on fecal samples at the end of the study. Receiver operating curves for diet classification (estimated by leave-one-out cross-validation). mOTUs refer to metagenomics OTUs [66]. **c** CAZy enzyme classes which show a differential response to the diet change (HPLC vs. LPHC). Shown is the ratio between pre-intervention and post-intervention samples (subjects where both samples were below the detection limit were removed from the analysis) (*p < 0.05, **p < 0.01, ***p < 0.001; Gehan’s two-sided test after multiple hypothesis correction)

Some taxa became detectable or undetectable in response to diet (the detection limit is ca. 2·10^−5^ in relative abundance). For example, *Lactobacillus ruminis* was not detected in any of the HPLC-fed dogs, even though it was present in 22% of the samples taken after baseline diet and was detected in 59% of LPHC-fed dogs (*p* = 8·10^−6^, Fisher’s exact test after Bonferroni correction; see Fig. 3a; Additional file 16: Figure S10). This is consistent with previous genome-based suggestions that this immuno-modulatory microbe may have an advantage in utilizing complex carbohydrates as a carbon source [49]. On the other hand, both *Intestinibacter bartlettii* and the entire *Streptococcus* genus are more frequently detected in dogs on the HPLC diet compared to both Base and LPHC. These strong prevalence effects suggest that these species may be amenable to modulation with prebiotics or with foods that selectively suppress taxa. One possibility for this increased prevalence may be how higher protein content directly advantages proteolytic fermenters or species which benefit indirectly from their metabolism in turn. Future, more detailed annotation of metabolic potential following from gut microbiome genes will allow comprehensive testing whether this effect explains the taxonomic changes observed under HPLC.

To identify major changes in functional composition, we linked the genes in the catalog to KEGG and CAZy enzyme classes and obtained functional profiles of the metagenomes [27]. The strength of the functional signals is exemplified by a penalized logistic regression classifier (see the “Methods” section; [50]) that can, based on either the functional or taxonomic profile of a sample, predict the diet which the dog was placed on (estimated by leave-one-out cross-validation; see Fig. 3b).

Of the genes that changed abundance in response to diet, five CAZy enzyme classes showed the strongest signal (Gehan’s test for doubly censored data [51], at a false discovery rate of 5%; Fig. 3c). Four glycohydrolase classes (GHs) become less abundant in the HPLC-fed dogs, which is consistent with these enzymes being involved in the metabolism of complex carbohydrates, while glycosyltransferase 6 (GT6) is more abundant in the guts of HPLC-fed dogs. Although the function of this ubiquitous enzyme in bacteria is still unclear [52], glycosyltransferases (GTs) in general catalyze formation of many different types of glycoproteins with important roles in cell-to-cell communication and recognition, thus perhaps utilizing or recycling carbohydrates.

**Fig. 4:**
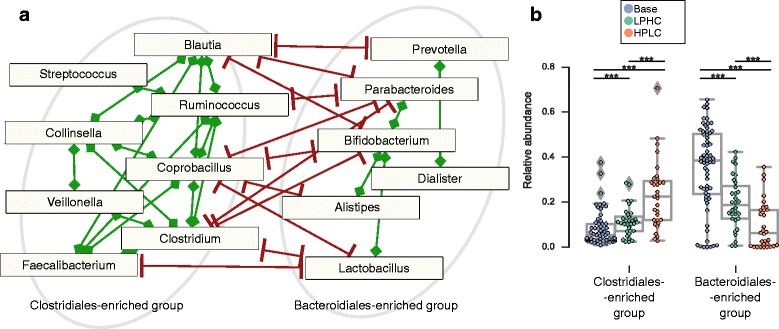
Analysis of co-abundance of genera. **a** Network of co-abundant genera (FDR of 5%, evaluated with sparCC; Spearman *r* > 0.5 or < − 0.5). Highlighted are two groups, one composed mainly of Clostridiales, the second of Bacteroideales. Green lines denote positive correlations, red lines negative ones. **b** Relative abundance of the Clostridiales-enriched and the Bacteroideales-enriched groups in each of the three diets studied (Base, HPLC, LPHC)

We subsequently identified functionally interacting species by searching for co-abundant taxa across the dog samples and found two large groups of microbial genera which have significantly correlated gut abundances (Spearman *r* > 0.5 in absolute value, statistical significance tested with sparCC [53], FDR set at 5%), within one group, but are anticorrelated in abundance with those of the other (Fig. 4). The first group, more abundant in dogs fed the HPLC diet, consists mainly of genera in the Clostridiales order, while the second one is enriched for Bacteroidiales. In mice, a decrease in Clostridiales was accompanied by an increase in Bacteroidiales in response to induced inflammation [54], while increased Clostridiales and decreased Bacteroidetes have been reported in response to high-fat and high-sucrose diets [55]. As discussed above, better resolution in metabolic annotation of gut microbial genomes and metagenomes may allow testing to what extent direct diet effects such as higher nutrient availability for different fermenters drive these compositional changes.

## Conclusions

Here, we present a dog gut microbiome gene catalog, which provides over 1 million taxonomically and functionally annotated genes and can serve as a resource for future studies. Together with the taxonomic census, our study of two dog breeds should provide a baseline for expanding dog microbiome research to other breeds, biogeography, and variation of living conditions (e.g., pets and feral dogs), thus disentangling the respective contribution of these factors to structuring the microbiome.

The structural and functional similarity of the dog microbiome to the human one implies that, as human studies are predictive of results in dogs, dog studies may be predictive of results in humans. Thus, dog studies provide a double benefit: for dogs directly and for their potential to generalize to humans. We illustrate this by a controlled dietary intervention study, in which we were able to reproduce high-level diet effects reported in other mammals, while also uncovering the higher responsiveness of the microbiome of overweight/obese dogs. This is a hypothesis to be tested in future human diet intervention studies.

## Methods

### Dog rearing

Sixty four dogs (32 Labrador retrievers and 32 beagles) were selected for an 8-week feeding study (Fig. 2a). Physical characteristics of the dogs were described in details in Additional file 1: Table S1. Dogs in each breed were divided according to their body fat percentages into two body condition groups: overweight or obese (OW) and lean or normal (LN). In the first 4 weeks, all dogs were on the commercially available Base diet. In the second 4 weeks, dogs in each breed and body condition group were first randomized by age, sex, and body fat percentage into two dietary intervention groups, HPLC and LPHC, and were switched to their assigned diets. Fecal samples were collected and body fat percentages were measured using dual energy X-ray absorption (DEXA, GE Lunar DPXα with EnCore 2011 software) after 4 weeks on the BASE diet and 4 weeks on the intervention diets.

A staggered start with two cohorts of dogs was implemented, the second cohort beginning 1 month after the first. Dogs in each body condition and dietary group were randomly assigned to the two cohorts by breed, age, and sex. Dogs were fed to maintain their body weights and were weighed weekly. The amount of food offered to a dog was adjusted by 5% if its body weight changed by 5% over its initial body weight. The maintenance energy requirement (MER) was estimated using the equation: MER = 139 × BW^0^· [56] (kilocalories), where BW is body weight in kilograms. Dogs had free access to the playgrounds and socialization activities. The OW dogs were fed to reduce their body weight after the study.

### Diets

All diets were formulated to be nutritionally complete and to comply with the guidelines of the Association of American Feed Control Officials and were manufactured by the Nestlé Purina PetCare Company. All diets contained animal protein as a primary protein source. The protein level in the HPLC diet was adjusted by replacing grains with plant protein. The macronutrient contents and energy densities of the three diets, BASE, HPLC, and LPHC, are listed in Additional file 6: Table S6.

### Sample collection and fecal DNA extraction

Canine fecal samples were collected within 15 min of defecation and immediately frozen at − 80 °C. The PowerFecal DNA Isolation Kit (MO BIO Laboratories, Inc., Carlsbad, CA, USA) was used to isolate fecal genomic DNA, following manufacturer’s protocol. DNA quantification was performed using PicoGreen® assay (Thermo Fisher Scientific, Waltham, MA, USA).

### Library preparation and Illumina sequencing

The library preparation was performed following the recommended Nextera XT protocol from Illumina. (Nextera XT DNA Library Preparation Guide, Part # 15031942 Rev. E January 2015, Illumina), with modifications for normalization and pooling as described below.

The library preparation workflow starts with a tagmentation (tag and fragmentation) of the genomic DNA followed by the samples barcoding in dual index (Nextera XT Index Kit v2 from Illumina) and an amplification (12 PCR cycles). After PCR, the library was purified using AMPure beads (Beckman Coulter) on a Sciclone robotic platform from Perkin Elmer. The quality and quantity of each library were evaluated using the LabChip GX Touch (capillary electrophoresis method from Perkin Elmer).

Libraries were pooled based on the molarity calculated by the LabChip GX Touch. The equimolar pool was assembled using the Hamilton robot and was controlled by a MiSeq run v2 50 cycles to be sure that each library clustered properly before sequencing on the HiSeq. The sequencing was performed on a HiSeq 2500 using v4 chemistry. The pool was loaded onto three flow cells with pair end reads of 125 bases and dual indexing (8 bases for each index).

Sequencing was performed on a HiSeq 2500 with the pool loaded onto three flow cells using v4 chemistry PE125 and dual indexing with (8 bases used for each index).

### Building a gene catalog

The gene catalog was built as previously described [27, 57]: after trimming and filtering (by removing reads which match either the canine reference genome or the human reference), contigs were assembled for each sample separately using SOAPDenovo2 [58]. Genes were then predicted on these reads with MetaGeneMark [59]. These genes were then clustered at 95% identity to form the final catalog using CD-HIT [60]. All 129 metagenomes were included in building the gene catalog. Pre- and post-dietary intervention samples from the same dog were treated as separate samples and processed independently.

### Taxonomic and functional annotation of genes and abundance estimations

For taxonomic annotation, we used the dual-BLAST least common ancestor approach [61] using Diamond as an alternative to BLAST [62]. Briefly, using Diamond, for each gene, we searched for homologs in the Uniprot database (version 2016_07). If no hits with e-value 10^−5^ or less were found, the result was “no hit.” Otherwise, the matched Uniprot region was used as input to a second homolog search against Uniprot and all hits whose e-value is equal or less than the original e-value were recorded. This constitutes the homolog neighborhood of the initial query gene. We assign to the query gene the least common ancestor of this neighborhood. This analysis was performed with the Jug scripts [63] in the taxonomic directory of the Supplementary Source Code.

Functional annotation of genes was performed using MOCAT2, which was also used to generate abundance profiles [27]. KEGG orthologous (KO) groups were filtered to include only KOs which were used in the annotation of prokaryotic species (thus removing any spurious hits to Eukaryotes). KEGG modules were similarly filtered to exclude modules which refer to more than 50% of non-prokaryotic KEGG. This filtering was performed with the Jug scripts in the prok-kos directory of the Supplementary Source Code.

### Strain-level SNP analysis

Single-nucleotide polymorphism (SNP) variation was quantified over representative specI genomes [64], using an approach previously described in [65] implemented by metaSNV [34]. Briefly, samples that had enough coverage over a given genome (5× vertical and 80% horizontal) were compared using a manhattan distance on the allele frequencies of the observed SNPs. This distance was normalized by the number of examined SNPs so that identical variation profiles have a distance of 0 and the ones that are completely distinct, a distance of 1.

Principal coordinate analysis on the distance matrices obtained as above was used to illustrate the variation space of bacterial species and show that dog samples are more closely related to other dog samples than they are to human subjects. This analysis is implemented by the Figure-PCoA_boxplots.py script.

### mOTUs construction and annotation

To determine the total microbial species composition of each stool sample, for both taxa previously identified and those yet to be isolated and characterized, mOTU-LGs were constructed as described by [66]. In brief, a non-redundant database of single copy marker genes (MGs), extracted from reference genomes as well as those assembled from canine and human samples, were clustered at gene-specific species-level identity cut-offs [64]. mOTU linkage groups (mOTU-LGs; all MGs belonging to a single microbial species) were produced by correlating MG abundances across all canine samples. In total, 228 mOTU-LGs were formed, containing between 2 and 10 MGs with an FDR of 0.02, these on average explained over 98% of total microbial abundance in a sample. mOTUs were annotated through BLAST of the assembled MGs against reference genome MGs using a last common ancestor approach; the reference annotation at each taxonomic level was migrated over to the mOTU-LG only when there was 100% agreement between all MG’s best hit results. For a mOTU-LG to be annotated to the species level, 100% agreement was required between top hits as well as an identity exceeding the MG clustering cut-off. mOTU-LG abundances were inferred from base-scaled read mappings standardized to the total length of the genes making up the linkage group. The relative abundance detection limit was estimated for each sample as the relative abundance of the least-abundant mOTU-LG. The global detection limit was the highest value (i.e., least sensitive) of all samples. This analysis was performed with the fetchMG tool [64] and the scripts in the motus directory of the Supplemental Software package.

### Statistical analyses of the microbiome profiles

Principal coordinate analyses were performed on log-transformed data using the Bray-Curtis distance. Comparisons between compositional shifts were computed based on the original distance matrix and compared using the two-tailed Wilcoxon test.

Differences in impact between diets under consideration were evaluated using the two-sided Gehan test [51]. Log-ratios were computed between the post-intervention and the baseline samples. A pseudo-count was added to each measurement (1/10th the value of the lowest non-zero detection). However, if the baseline sample was below detection limit, the ratio was considered right-censored, and if the post-intervention sample was below detection limit, the ratio was considered left-censored. When both pre- and post-intervention samples were below detection limit, the sample was not taken into consideration. Multiple hypothesis testing was corrected using the two-stage Benjamini-Hochberg method [67].

Co-abundance statistical abundances were performed using sparCC to estimate empirical *p* values [53].

Predictions of diet based on taxonomic/functional profiles were performed using a penalized logistic regression classifier, using inner cross-validation for the estimation of the hyperparameters, as implemented by scikit-learn [56]. Prior to model fitting, features were normalized by ranks as done previously [68].

The analysis in this subsection was performed using the scripts in the analyses directory.

## Additional files

Additional file 1: **Table S1.** Properties of the dogs. (XLSX 15 kb)

Additional file 2: **Table S2.** Basic statistics of the metagenomics data: number of base pairs and reads per sample. (XLSX 12 kb)

Additional file 3 **Table S3.** Gene annotation statistics. (XLSX 6 kb)

Additional file 4 **Table S4.** Phylum-level relative abundances of the microbiota. (XLSX 5 kb)

Additional file 5: **Table S5.** Spearman correlation and SparCC-derived *p* value for all nodes in the co-abundance correlation network analyzed. (XLSX 7 kb)

Additional file 6: **Table S6.** Nutritional content of the 3 diets used in this study. Values shown refer to percentage (%) by weight. (XLSX 4 kb)

Additional file 7: **Figure S1.** Gene accumulation curve for dog, pig, mouse, and human gut microbiomes. (PDF 1676 kb)

Additional file 8: **Figure S2.** (a) Distance between samples from multiple hosts (and from two separate human cohorts as a control) measured by abundance-weighted Jaccard distance (b) overlap in detected (named) genera (genera with prevalence > 1%) [number of genera]. (PDF 49 kb)

Additional file 9: **Figure S3.** Mapping rates of human reads to the gut gene catalogs of the four mammalian hosts considered (humans, mice, dogs, and pigs). (PDF 1848 kb)

Additional file 10: **Figure S4.** Gene content overlap between human, dog, pig, and mouse catalogs after downsampling the larger catalogs down (cf. Fig. 1d). (a) Genes were randomly selected; (b) Genes were selected as to cover 90% of the abundance in metagenomes (on average). (PDF 21 kb)

Additional file 11: **Figure S5.** Principal coordinate analysis of SNP profile of species whose abundance is large enough to reliably profile in dog and human stool metagenomes. (PDF 29 kb)

Additional file 12: **Figure S6.** Distance boxplots of the samples in the 3 diets using Bray-Curtis divergence on log-normalized data (corresponding to Fig. 2c) (PDF 25 kb)

Additional file 13: **Figure S7.** (a) *left*: Principal coordinate analysis using weighted Unifrac distance [37]; *right*: corresponding distance boxplot (b) *left*: Principal coordinate analysis using weighted PINA distance [38]; right: corresponding distance boxplot (*p < 0.05; **p < 0.01; ***p < 0.001: Mann-Whitney-Wilcoxon two-tailed test). (PDF 301 kb)

Additional file 14: **Figure S8.** (a) Firmicutes:Bacteroidetes ratio at the end of the Base feeding period (no significant difference) (b) Firmicutes and Bacteroidetes relative abundances (c) Firmicutes:Bacteroidetes ratio (*p < 0.05; **p < 0.01; ***p < 0.001: Mann-Whitney-Wilcoxon two-tailed test). (PDF 350 kb)

Additional file 15: **Figure S9.** (a) *Bacteroides* and *Prevotella* relative abundances as a function of the diet. (b) ratios between the two genera (*p < 0.05; **p < 0.01; ***p < 0.001: Mann-Whitney-Wilcoxon two-tailed test). (PDF 259 kb)

Additional file 16: **Figure S10.** Prevalence change split by experimental cohorts (cf. Fig. 3a). (PDF 42 kb)
