# Supplementary figures and images for: Similarity of the dog and human gut microbiomes in gene content and response to diet
Source: Microbiome. 2018 Apr 19;6:72. doi: 10.1186/s40168-018-0450-3 (PMC5907387; doi:10.1186/s40168-018-0450-3)

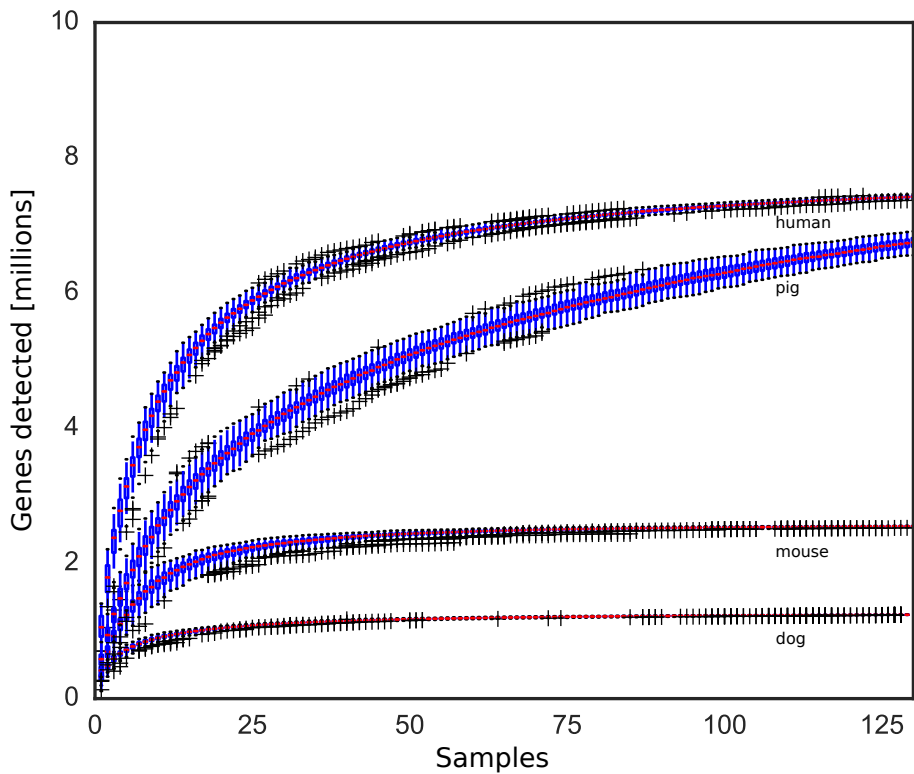

Supplement: Supplementary file 7 — Figure S1. Gene accumulation curve for dog, pig, mouse, and human gut microbiomes. (PDF 1676 kb) [file 40168_2018_450_MOESM7_ESM.pdf]

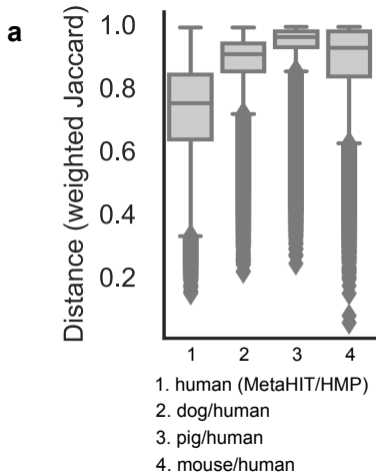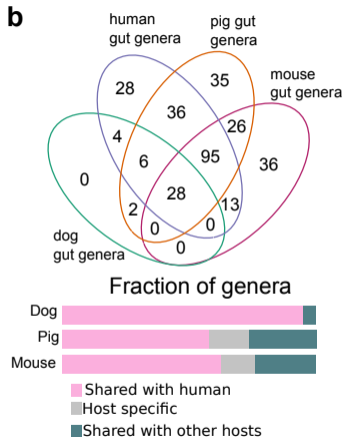

Supplement: Supplementary file 8 — Figure S2. (a) Distance between samples from multiple hosts (and from two separate human cohorts as a control) measured by abundance-weighted Jaccard distance (b) overlap in detected (named) genera (genera with prevalence > 1%) [number of genera]. (PDF 49 kb) [file 40168_2018_450_MOESM8_ESM.pdf]

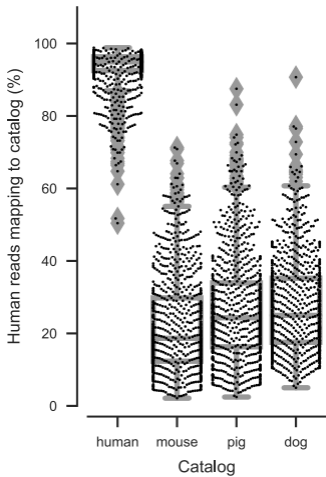

Supplement: Supplementary file 9 — Figure S3. Mapping rates of human reads to the gut gene catalogs of the four mammalian hosts considered (humans, mice, dogs, and pigs). (PDF 1848 kb) [file 40168_2018_450_MOESM9_ESM.pdf]

**a**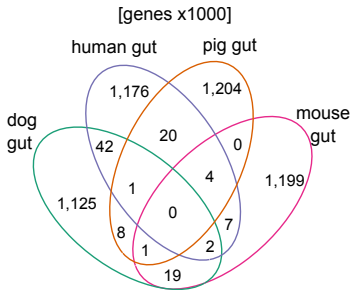**b**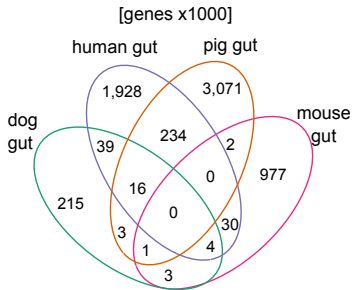

Supplement: Supplementary file 10 — Figure S4. Gene content overlap between human, dog, pig, and mouse catalogs after downsampling the larger catalogs down (cf. Fig. 1d). (a) Genes were randomly selected; (b) Genes were selected as to cover 90% of the abundance in metagenomes (on average). (PDF 21 kb) [file 40168_2018_450_MOESM10_ESM.pdf]

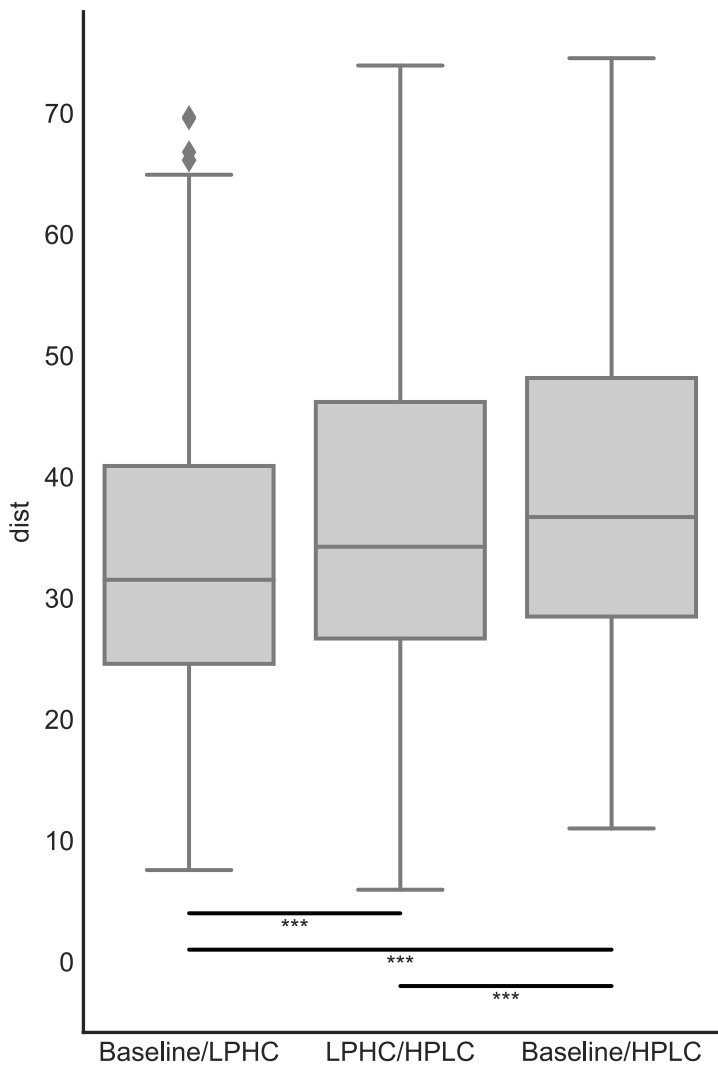

Supplement: Supplementary file 12 — Figure S6. Distance boxplots of the samples in the 3 diets using Bray-Curtis divergence on log-normalized data (corresponding to Fig. 2c) (PDF 25 kb) [file 40168_2018_450_MOESM12_ESM.pdf]

**a**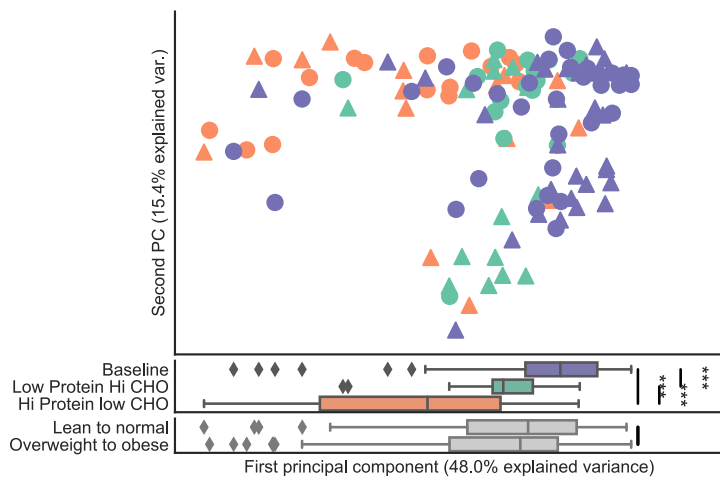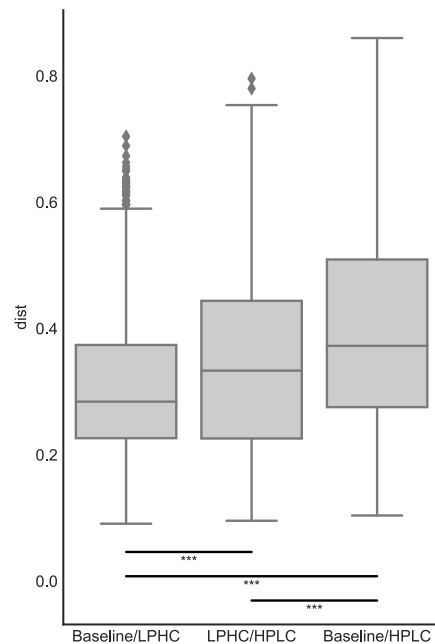**b**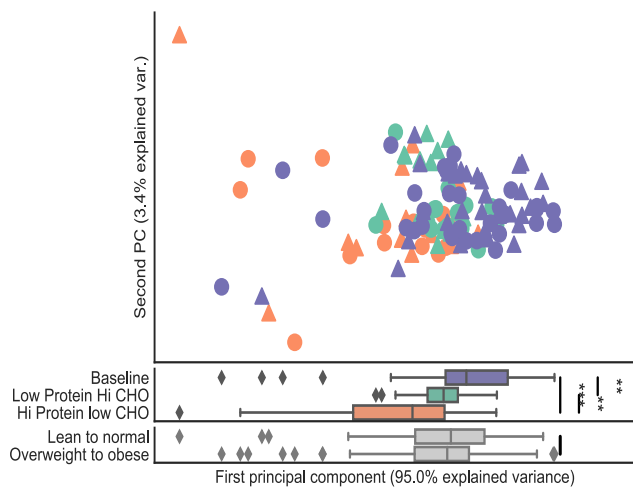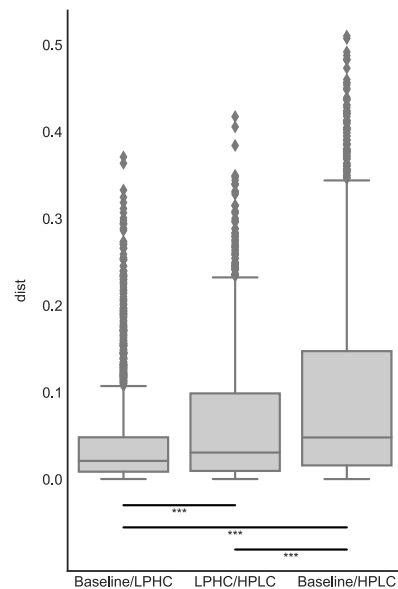

Supplement: Supplementary file 13 — Figure S7. (a) left: Principal coordinate analysis using weighted Unifrac distance [37]; right: corresponding distance boxplot (b) left: Principal coordinate analysis using weighted PINA distance [38]; right: corresponding distance boxplot (*p < 0.05; **p < 0.01; ***p < 0.001: Mann-Whitney-Wilcoxon two-tailed test). (PDF 301 kb) [file 40168_2018_450_MOESM13_ESM.pdf]

**a**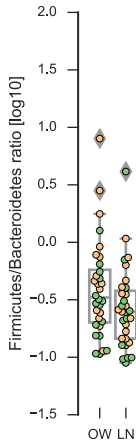**b**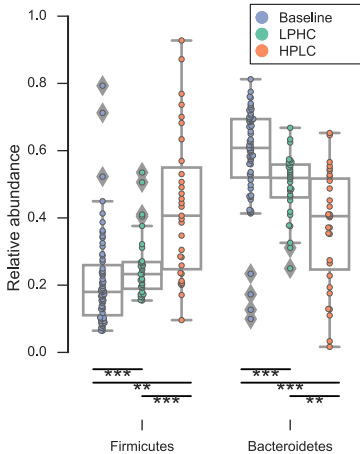**c**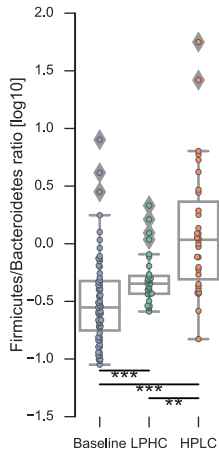

Supplement: Supplementary file 14 — Figure S8. (a) Firmicutes:Bacteroidetes ratio at the end of the Base feeding period (no significant difference) (b) Firmicutes and Bacteroidetes relative abundances (c) Firmicutes:Bacteroidetes ratio (*p < 0.05; **p < 0.01; ***p < 0.001: Mann-Whitney-Wilcoxon two-tailed test). (PDF 350 kb) [file 40168_2018_450_MOESM14_ESM.pdf]

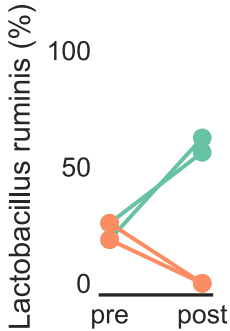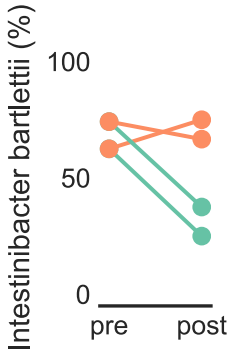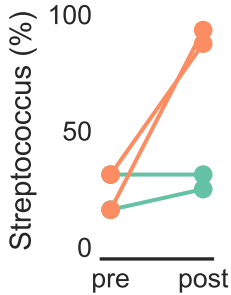

Supplement: Supplementary file 16 — Figure S10. Prevalence change split by experimental cohorts (cf. Fig. 3a). (PDF 42 kb) [file 40168_2018_450_MOESM16_ESM.pdf]
