# Supplementary material for: Similarity of the dog and human gut microbiomes in gene content and response to diet
Source: Microbiome. 2018 Apr 19;6:72. doi: 10.1186/s40168-018-0450-3 (PMC5907387; doi:10.1186/s40168-018-0450-3)
Supplement: Supplementary file 15 — Figure S9. (a) Bacteroides and Prevotella relative abundances as a function of the diet. (b) ratios between the two genera (*p < 0.05; **p < 0.01; ***p < 0.001: Mann-Whitney-Wilcoxon two-tailed test). (PDF 259 kb) [file 40168_2018_450_MOESM15_ESM.pdf]

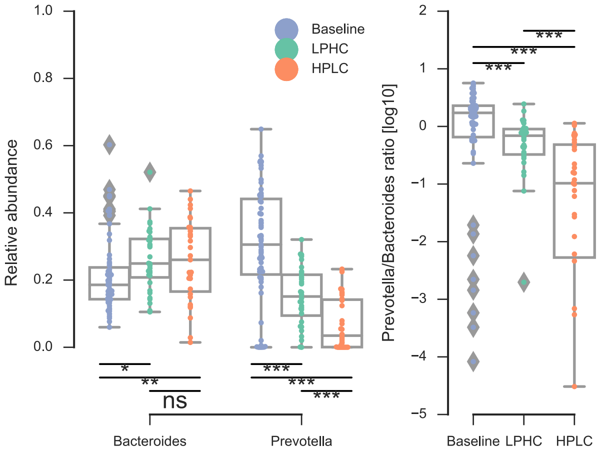

Baseline
LPHC
HPLC
Relative abundance
1.0
0.8
0.6
0.4
0.2
0.0
*
**
ns
***
***
***
Bacteroides
Prevotella
Prevotella/Bacteroides ratio [log10]
2
1
0
−1
−2
−3
−4
−5
***
***
***
Baseline
LPHC
HPLC
