# Supplementary material for: Similarity of the dog and human gut microbiomes in gene content and response to diet
Source: Microbiome. 2018 Apr 19;6:72. doi: 10.1186/s40168-018-0450-3 (PMC5907387; doi:10.1186/s40168-018-0450-3)
Supplement: Supplementary file 11 — Figure S5. Principal coordinate analysis of SNP profile of species whose abundance is large enough to reliably profile in dog and human stool metagenomes. (PDF 29 kb) [file 40168_2018_450_MOESM11_ESM.pdf]

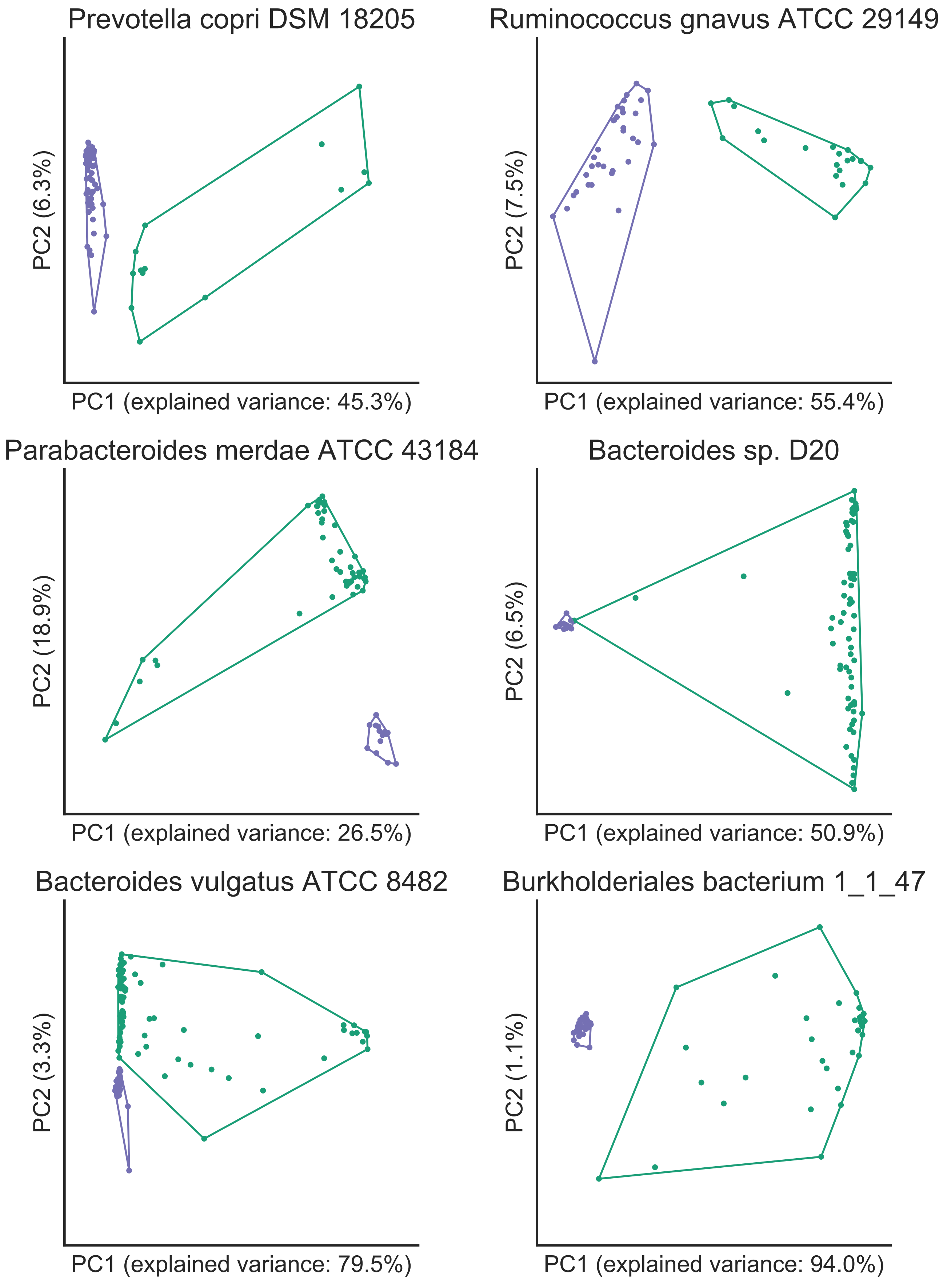
Prevotella copri DSM 18205
PC2 (6.3%)
PC1 (explained variance: 45.3%)
Ruminococcus gnavus ATCC 29149
PC2 (7.5%)
PC1 (explained variance: 55.4%)
Parabacteroides merdae ATCC 43184
PC2 (18.9%)
PC1 (explained variance: 26.5%)
Bacteroides sp. D20
PC2 (6.5%)
PC1 (explained variance: 50.9%)
Bacteroides vulgatus ATCC 8482
PC2 (3.3%)
PC1 (explained variance: 79.5%)
Burkholderiales bacterium 1_1_47
PC2 (1.1%)
PC1 (explained variance: 94.0%)
